# Miscellany

**Published:** 1901-03

**Authors:** 


### MISCELLANY.

The Surgeon-General, U. S. Army, announces that as a result of the reorganisation of the army by act of congress, approved February 2, 1901, there are 129 original vacancies as assistant surgeon to be filled.

The prescribed age limit of candidates will be rigidly adhered to and it will be useless for those who are more than twenty-nine years of age to apply, unless they have had previous service, as mentioned in the circular. The requirement of hospital experience or its equivalent in private practice will also be insisted upon. Hospital work as a student will not be regarded as fulfilling this requirement; experience as a physician after graduation is what is meant. Two years of private practice will be considered an equivalent of one year's hospital experience.

Army Medical Boards will be convened at an early day in Washington and in San Francisco, and examinations will be begun as soon as possible. Copies of a circular, giving adequate information to candidates may be obtained upon application to the Surgeon-General, U. S. Army, Washington, D.C.

The New York Pharmacal Association, the makers of lactopeptine, liquid peptonoids and hemaboloids are engaged in compiling and arranging a 64-page book, entitled Facts and Figures, Medical and otherwise, from the Census of 1900 and other reliable sources. Fully one-half of this volume (32 pages) is made of full-page colored lithographic maps and schematic diagrams, illustrating in a thoroughly clear, concise and graphic manner the most interesting and important subject matter.

Besides much interesting and important statistics thus graphically illustrated of a general character,there is much of medical and climatological interest, such as maps and diagrams,illustrative of the comparative mortality of the various infectious diseases in the twelve principal cities of the United States; diphtheria mortality of New York, Massachusetts, Philadelphia, England, London, Chicago, Berlin, Paris, etc., showing graphically the pre- and post-antitoxin death-rate; mortality from anesthetics; ratio of deaths to inhalations; maps showing variations of altitude, sunshine, rainfall, etc., of different sections of the United States; schematic design, showing comparative elevation above sea-level of the principal health resorts, with climatological and meteorological data relating to each. Other attractive charts represent the division of the population of each state and territory as regards city and country dwellers; color, race, etc.

The publishers intend to present this handsome, artistic, instructive and unusually expensive souvenir to their friends in the medical and dental professions, and will be pleased to receive and file requests for same, mentioning this journal.

